# Prevalence and characteristics of apical aneurysm on cardiovascular magnetic resonance in patients with hypertrophic cardiomyopathy

**DOI:** 10.1186/1532-429X-16-S1-P304

**Published:** 2014-01-16

**Authors:** Eun Kyoung Kim, Sang-Chol Lee, Hye Bin Gwag, Sung-A Chang, Sung-Ji Park, Yeon Hyeon Choe, Sung-Mok Kim, Seung Woo Park

**Affiliations:** 1 Cardiology, Department of Medicine, Cardiovascular Imaging Center, Samsung Medical Center, Sungkyunkwan University School of Medicine, Seoul, Korea, Republic of; 2 Radiology and Center for Imaging Science, Cardiovascular Imaging Center, Samsung Medical Center, Sungkyunkwan University School of Medicine, Seoul, Korea, Republic of

## Background

Hypertrophic cardiomyopathy (HCM) with apical aneurysm (AAn) is associated with considerable morbidity and mortality. However, the real incidence of AAn tends to be underrecognized due to the poor visualization of left ventricular (LV) apex with echocardiography. This study sought to investigate the exact incidence and associated manifestations of AAn using cardiovascular magnetic resonance (CMR) in patients with HCM.

## Methods

A total of 350 consecutive patients diagnosed with HCM (mean age 54 ± 12, 278 males) underwent CMR and echocardiography. We divided the subjects into 4 phenotypes according to the location of hypertrophic segment; asymmetrical septal hypertrophy (ASH), apical, concentric and septal/apical type. On CMR, the LV volumetric parameters were measured, and the amount of LGE was calculated with gray-scale thresholds of 6 SD above the mean signal intensity for normal remote myocardium. Echocardiographic evaluations included left atrial volume index, mitral inflow pattern, tissue Doppler of mitral annulus and LV dimension.

## Results

The prevalence of AAn on CMR was 14.3%, which was significantly high compared to previously reported data. AAn was detected in all groups of HCM regardless of type (16.8% in ASH type, 15.3% in apical type, 17.9% in concentric type, and 9.1% in septal/apical type of HCM). Clinical manifestations and LV volumetric parameters on CMR did not differ between the HCM patients with and without AAn. The frequency and the amount of late gadolinium enhancement were not different between two groups (frequency; 94% vs. 93.3%, p = 1.00, extent; 11.7 ± 8.9 vs. 13.0 ± 10.3, p = 0.43).

## Conclusions

The incidence of AAn in HCM patients was far higher than it was reported previously. Regardless of presence of AAn, initial manifestations and associated morphology of LV were similar. This means that adverse clinical outcomes in HCM patients with AAn may be a long-range problem which arises from secondary myocardial changes due to AAn.

## Funding

None.

